# Carbon-Free Solution-Based Doping for Silicon

**DOI:** 10.3390/nano11082006

**Published:** 2021-08-05

**Authors:** Sebastiano Caccamo, Rosaria Anna Puglisi

**Affiliations:** Istituto per la Microelettronica e Microsistemi, Consiglio Nazionale delle Ricerche, 95121 Catania, Italy; caccamo.seby@gmail.com

**Keywords:** molecular doping, semiconductors, semiconductor doping, metallurgical junction, electrical properties, carbon-free

## Abstract

Molecular doping is a method to dope semiconductors based on the use of liquid solutions as precursors of the dopant. The molecules are deposited on the material, forming a self-ordered monolayer that conforms to the surfaces, whether they are planar or structured. So far, molecular doping has been used with precursors of organic molecules, which also release the carbon in the semiconductor. The carbon atoms, acting as traps for charge carriers, deteriorate the doping efficiency. For rapid and extensive industrial exploitation, the need for a method that removes carbon has therefore been raised. In this paper, we use phosphoric acid as a precursor of the dopant. It does not contain carbon and has a smaller steric footprint than the molecules used in the literature, thus allowing a much higher predetermined surface density. We demonstrate doses of electrical carriers as high as 3 × 10^15^ #/cm^2^, with peaks of 1 × 10^20^ #/cm^3^, and high repeatability of the process, indicating an outstanding yield compared to traditional MD methods.

## 1. Introduction

The most recent semiconductor roadmap requires low voltage and low power devices while maintaining high performance and low manufacturing costs. As a solution to these technological requirements, it has been proposed to fabricate the devices, now reaching nanometric sizes, on structures with 3D geometries. In this context, the standard doping methods exhibit strong limitations: (i) they do not allow the doping to be carried out in such a way that it follows the 3D nanostructured surfaces, the so-called ‘conformal’ doping; (ii) they use expensive equipment and materials; (iii) they lead to the formation of structural defects within the semiconductor, only partially solved by subsequent heating, or uncontrolled formation of precipitates; (iv) finally, they exhibit difficult manageability in the realization of specific applications such as ultra-thin junctions (under 10 nm).

Recently, an alternative method of doping has been proposed based on the use of liquid solutions, the Molecular Doping (MD) [1,2,3,4,5,6,7]. The dopant precursor is in liquid form, and the semiconductor (e.g., silicon, germanium, or gallium arsenide) is immersed in the solution. During the immersion process, the molecule containing the dopant atom is deposited on the surface of the material. Being the precursor of the dopant in liquid form and coming into direct contact with the surface to be treated, MD allows conformal doping, i.e., it follows the surface of complex structures, such as nanostructured, porous, or hollow ones. In this method, moreover, the surface density of the dopant depends on how many molecules are initially deposited on the silicon surface. Since they bind to it, in order to form a compact self-assembled monolayer, this density depends on the steric footprint of the molecule and is therefore predetermined once the molecule is designed. This represents a further advantage of MD compared to traditional methods, based on approaches that envisage the adhesion/introduction of the dopant atom based on the statistical position of its arrival. The mechanism lying underneath the binding process between the molecular precursor monolayer and the semiconductor surface is the double bond breakage between the oxygen and the dopant atom, with the subsequent formation of a covalent bond between the oxygen and the substrate atoms [8].

Despite the promising results, however, a determining aspect that has prevented a wide and extensive development of the MD in the industrial field is linked to the problem that the atoms constituting the molecule can diffuse together with the dopant atoms inside the sample. The methods proposed in the literature so far use organic molecules, such as esters or alcohols, as dopant precursors. In Ref. [1], the authors doped silicon (Si) wafers by firstly treating the Si surfaces with a dopant dissolved in mesitylene (the dopant being allylboronic acid pinacol ester for p-type doping, and diethyl-1-propylphosphonate, known as DPP, for n-doping), and subsequently annealed the material to diffuse the dopant atoms in the surface and achieve n+/p-n or p+/n- ultra-shallow junctions. In the method explained in [9], the solution is made of tetraethylene glycol dimethyl ether (tetraglyme) and a dopant precursor such as DPP or allylboronic acid pinacol ester.

When the precursor is decomposed to release the dopant atom to be diffused into the silicon, the carbon atoms that constitute the molecule are also released and can play a role from an electrical point of view within the Si. It is known indeed that carbon is identified as a benign contaminant for some devices, such as MOS and bipolar devices, but in others, such as high voltage diodes and transistors, at concentrations higher than 5 × 10^16^ cm^−3^, it forms structural defects and electronic deep traps in the Si bandgap, and consequently, it deteriorates the device’s electrical properties. Moreover, in the case of diffusion phenomena at low depths, about 2–3 nm, with concentrations higher than 1 × 10^21^ cm^−3^, it forms a thin layer of silicon carbide (SiC) resistant to chemical etching. Although this layer is confined to the surface, this has been identified as one of the limits of the technique [6,10]. Moreover, interstitial carbon, Ci, can bond with substitutional phosphorus, Ps, forming the pair Ci–Ps, with multiple deep energy levels that contribute to the P deactivation [11].

Rapid and extensive industrial exploitation has therefore raised the need for a method that can eliminate the carbon presence altogether. Some works in the literature have proposed the use of alternative precursors to the DPP to reduce the carbon impact, but always without eliminating it, providing further inconveniences which made the technological process less attractive [9,10,11].

Moreover, the step of dipping the semiconductor in an aqueous ambient temperature solution does not ensure any fine controllability of the dopant coating achieved in the doping process. Ref. [12] presents work using phosphoric acid dissolved in different organic solvents as well as deionized water. However, deionized water resulted in the silicon surface being hydrophobic. Instead, butanol as a solvent exhibited uniform sheet resistance over the wafer surface compared to other solvents. Moreover, a phosphorus silicate glass (PSG) layer is formed on the sample surface, which needs to be removed by a further expensive process.

Here we show how to overcome the limits of the literature approaches, by using a simple, reliable, efficient, eco-friendly, inexpensive way and completely carbon-free, to dope a semiconductor, even with complex, three-dimensional and hollow structures, producing high concentrations of dopant atoms and a deterministically controlled distribution. We chose phosphoric acid (PA) because its molecular structure is composed only of oxygen, hydrogen and phosphorus, and no carbon atoms are present [13]. We also demonstrate that PA is electrically more efficient than DPP. As in the case of the DPP, the phosphorus is bound to an oxygen atom through a double bond that is energetically favored for the formation of the bond with the silicon. One of the advantages of PA is that it is used in many foods and drinks as a mold and bacteria preservative or to improve the tangy taste. It is also used in dental cement, in the preparation of albumin derivatives and in the sugar and textile industries. Moreover, it is already widely used in microelectronics factories because it is used in the removal of silicon nitride, one of the most common compounds. Another very important advantage of PA is that it has a high selectivity with respect to silicon, also at high concentrations [14,15]. Furthermore, it can be diluted in water rather than in organic solvents such as mesitylene, used in the traditional MD method. This eliminates the carbon present in the solution. To test the efficacy of PA as a molecular precursor, we tested it on silicon, using methods like those of MD to exploit the low cost, simplicity, speed of process and conformality without the need to use expensive and dangerous equipment and materials for the environment and operators.

## 2. Materials and Methods

We developed a procedure to process the samples with the new methodology: the samples were p-type <111> Si samples of 1 × 1 cm^2^ in size, with an R = 1–10 Ω cm. We ultrasonically cleaned them with acetone, alcohol and finally water, each step for 5 min, to remove the organic and non-organic surface contaminants. Subsequently, we immersed them in hydrofluoric acid solution, HF, to remove the native SiO_2_ oxide naturally present on the Si surface. We then immediately immersed the samples in a 20% solution of phosphoric acid diluted in water and brought them to the boiling point of 120 °C for 2.5 h (for times ranging between 50 and 200 min). In other cases, the condition in which the solution was kept at room temperature was also explored to verify its effectiveness even in further simplified cases, together with several other dilutions. A group of samples, after the MD deposition with PA, were subjected to a cleaning treatment with water or acetone rinsing for 5 min to investigate the effects on the morphology and on the final electrical yield. Then the samples were heated to 1050 °C in a furnace in N_2_ ambient for times ranging between 20 and 500 s to diffuse the phosphorous in the Si matrix. We performed the specimen morphological characterization by Atomic Force Microscopy (AFM) measurements and by Spreading Resistance Profiling (SRP), which allows an advanced carrier profiling as a function of the sample depth.

## 3. Results and Discussion

**Figure 1:**
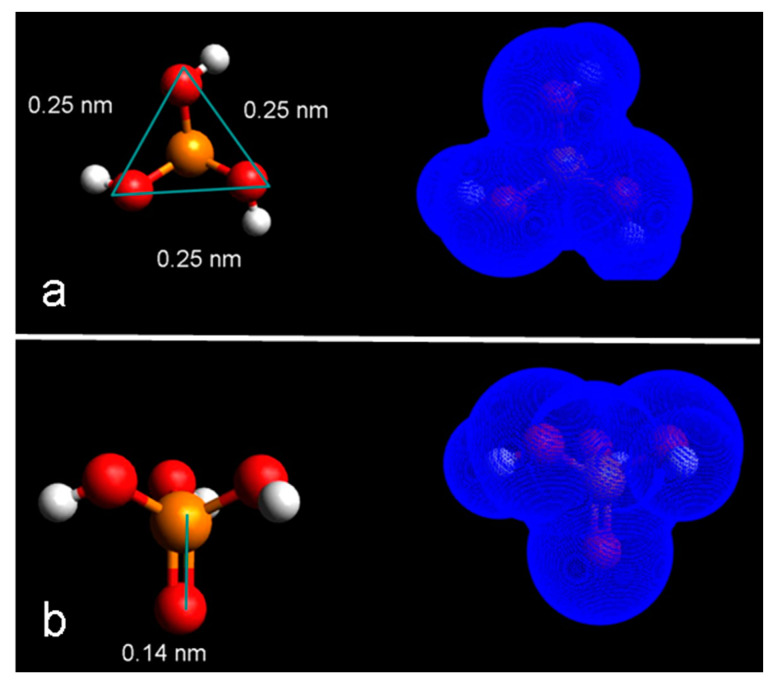
Avogadro modeling of the PA molecule structure (**a**,**b**) (**left**) with corresponding Van der Waals surfaces (**right**) obtained by using Avogadro software based on the universal force field approximation.

The main advantage of the proposed approach is related to the fact that the source molecule does not contain carbon in its composition. Moreover, since it is composed of fewer atoms with respect to the traditional molecules, it allows for an improved packing capability and, therefore, a higher initial dopant density. Figure 1 shows the modeling of the PA molecule structure (a,b) (left) with corresponding Van der Waals surfaces (right) obtained by using Avogadro software based on the universal force field approximation. The PA molecule maximum lateral extension (a), and the minimum lateral extension (b), have been calculated and are respectively 0.25 nm and 0.14 nm. The size of the DPP molecule, typically used in literature for this type of solution-based doping, is larger, exhibiting a maximum lateral extension of 0.9 nm and a minimum lateral extension of 0.7 nm [8]. This is a very important aspect because it ensures a higher packing effect during the self-assembling process, leading, therefore, to a higher molecular/dopant density on the Si surface after the deposition. In addition to the small steric footprint, PA also has a ratio phosphorus/[rest of atoms] equal to 1:8, more favorable than that of DPP, with a ratio equal to 1:28.

**Figure 2:**
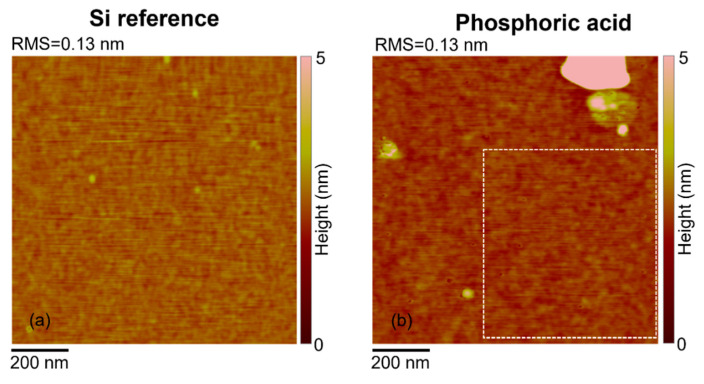
AFM maps taken on the reference Si wafer (**a**) and on the sample after the complete MD procedure: PA deposition followed by the cleaning and the diffusion annealing process (**b**). The measured RMS is 0.13 nm in both cases.

**Figure 3:**
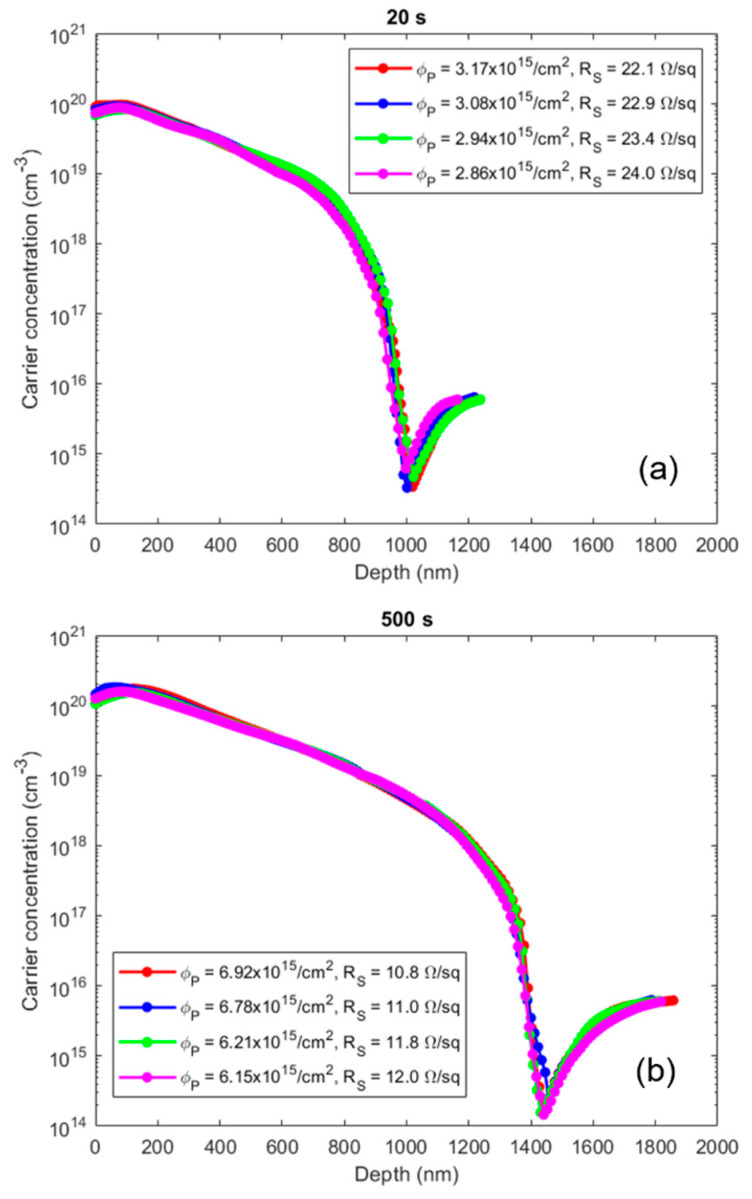
Comparison of the electrical results obtained by SRP analysis on samples treated with PA kept at 120 °C and subsequently annealed at 1050 °C for 20 s (**a**) and 500 s (**b**).

Figure 2 shows the AFM maps acquired on the reference Si wafer (a) and on the sample after the complete MD procedure, i.e., the PA deposition followed by the cleaning in water and the diffusion annealing process (b). The measured RMS is 0.13 nm in both cases, indicating that we are observing the Si surface and that the molecule has been dissolved, as expected. PA indeed is known to have a high selectivity with respect to Si, even at very high concentrations [14,15]. This indicates another important advantage of the procedure based on PA, the fact that the surface is kept intact after the whole doping process. Figure 3a shows the SRP results obtained on four different samples doped with PA and processed at the annealing temperature of 1050 °C for 20 s, after the MD deposition. The dose of carriers obtained by integrating the reported profiles is as high as 3 × 10^15^ #/cm^2^, indicating an outstanding yield compared to both traditional and MD methods [1,2,3,4,5,6,7]. The peak of the charge carriers is 1 × 10^20^ #/cm^3^. The curves are superimposed to demonstrate the high repeatability of the process.

A second process was performed by increasing the annealing time from 20 s to 500 s to investigate whether the spreading length of charge carriers observed in doped samples with PA can be modulated through the annealing time. As in the previous case, the samples were characterized by SRP, and the profiles were reported in Figure 3b. In this case, the profiles obtained are different from those scored for 20 s. In fact, the dose is about twice that obtained in the previous case, the Rs is half and the junction depth value is larger. The dose of carriers obtained is, in this case, equal to about 6.5 × 10^15^ #/cm^2^. The peak of the charge carriers is again 1 × 10^20^ #/cm^3^. The two results shown in Figure 3a,b demonstrate that the carrier profiles can be tuned by changing the annealing conditions as in the DPP case [16].

Comparing these results with those doped with DPP in mesitylene in terms of carrier dose and sheet resistance, the differences are evident: the dose in fact obtained in the case of PA is more than an order of magnitude greater than in the case of the DPP. Despite the same processing conditions (temperature, time and annealing ambient), we have observed different electrical properties, such as the dose, sheet resistance and diffusion lengths, in the samples doped by PA compared to the DPP doped samples. These differences could be ascribed to: (i) the change of the molecular precursor, (ii) to anomalous diffusion effects and/or to nonlinear diffusivity at high concentrations. The first explanation relates to the smaller dimensions of the phosphoric acid molecule compared to the DPP, leading, as discussed, to an initial higher dopant surface density after the molecule self-assembling on the Si surface. A further contribution could probably be due to the Si surface oxidation during the annealing process, which takes place in spite of the inert annealing conditions. The oxidation indeed produces a super-saturation of interstitial defects within the substrate that is responsible for a greater diffusion length [17,18].

As suggested in the literature, a further explanation is possible: interstitial carbon, Ci, can bond with substitutional phosphorus, Ps, forming the Ci-Ps complexes that contribute to the P deactivation [11] not present when PA is used. This hypothesis is supported in our data by the ICP-MS measurements (reported in Table 1 showing the dose of electrically active carriers obtained by SRP and the percentage of P as chemical species, for the DPP molecule and for the PA, indicating that the activation of the dopant is favored in the PA-based, carbon-free approach.

**Figure 4:**
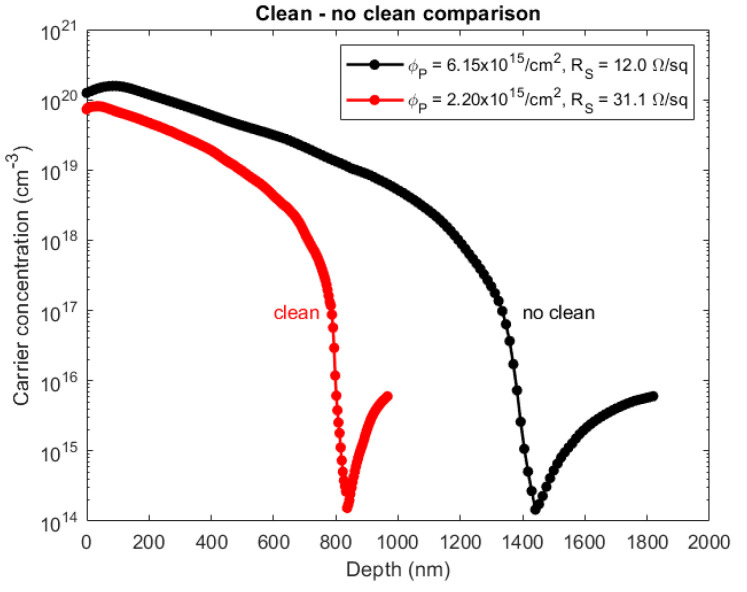
Electrically active carrier concentration as a function of depth on samples kept at 120 °C, cleaned after the PA deposition (red curve) or not (black curve) and subsequently annealed at 1050 °C for 500 s.

Figure 4 reports the comparison between the SRP profile obtained for the samples treated with PA deposition and annealed right after the deposition (black curve, ‘no clean’ case) and the samples rinsed in water before the annealing (red curve, ‘clean’ case). As it is possible to see, the cleaning procedure reduces the carrier concentration and dose; however, the electrical dose is as high as 2.20 × 10^15^ cm^−2^ and the sheet resistance as low as 31.1 Ω/sq.

**Figure 5:**
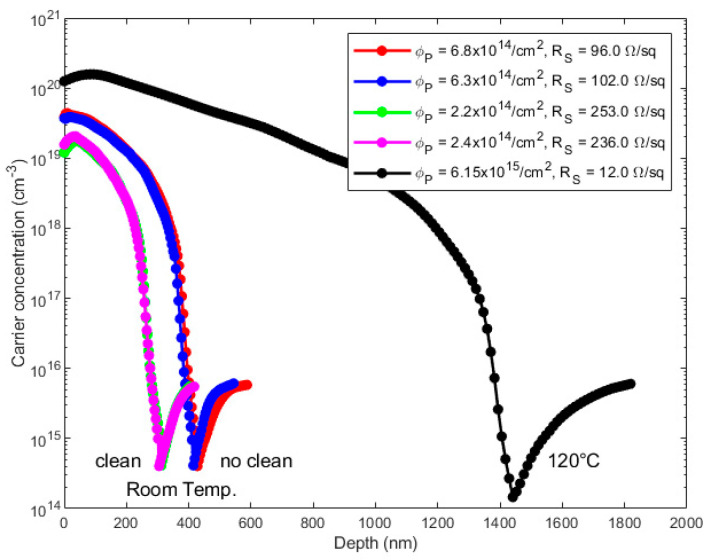
Electrical results on samples kept at room temperature, cleaned and not cleaned, compared to the reference case (black curve) obtained at 120 °C without cleaning. All the samples have been annealed at 1050 °C for 500 s.

Finally, Figure 5 illustrates the results obtained if the deposition of the PA molecule takes place at room temperature instead of at boiling conditions, compared to the reference case (black curve) obtained at 120 °C without cleaning. In all cases, we annealed the samples at 1050 °C for 500 s. As it is possible to see, with respect to the reference case, the low temperature leads to a loss in carrier dose of more than one order of magnitude, from about 6 × 10^15^ cm^−2^ to about 6 × 10^14^ cm^−2^, with a consequent increment in the sheet resistance. These results indicate that the grafting mechanism taking place between the molecule and the Si surface during the deposition performed at boiling temperature, as in the cases reported in Figure 3b, has a positive impact on the final electrical performances of the material if compared to the room temperature conditions.

## 4. Conclusions

In conclusion, we have demonstrated a method to effectively dope silicon based on the use of liquid solutions containing the dopant atoms in the molecules. We proposed phosphoric acid as a precursor of the dopant. The proposed molecule does not contain carbon, and this allows us to overcome the main limitation present so far for this type of solution-based doping. PA can also be dissolved in water, and this permits the elimination of organic solvents from which the carbon contaminant can arise too. Moreover, PA, as a dopant source, has a smaller steric footprint than the molecules used so far in the literature, thus allowing a much higher predetermined surface density. We demonstrate doses of electrical carriers as high as 6 × 10^15^ #/cm^2^ in the untreated samples and of about 3 × 10^15^ #/cm^2^ in the treated cases with peaks of 1 × 10^20^ #/cm^3^ and high repeatability of the process, indicating an outstanding yield compared to traditional MD methods. Using the temperature of 120 °C for the deposition of PA demonstrated the major benefit of providing higher carrier doses with respect to the room temperature conditions, for which the obtained dose was one order of magnitude smaller. PA is also cost-effective, it is currently used in the food industry and it is then less harmful than the typical materials/approaches used for the doping of semiconductors. The results demonstrate the potentiality of the method for rapid and extensive industrial exploitation, overcoming the previous MD drawbacks.

## 5. Patents

“Molecular doping”, Rosaria Anna Puglisi, Sebastiano Caccamo, Italian patent application No. 102019000006641 filed on 8 May 2019, International application No. PCT/IB2020/054377 filed on 8 May 2020.

## Figures and Tables

**Table 1:** Dose of electrically active carriers obtained by SRP and the percentage of P as chemical species.

Molecule	SRP (#/cm^2^)	P (%)
DPP	1.58 × 10^14^	1.7
PA	6.5 × 10^15^	43.4
